# Sex differences in physical performance, sleep, and psychological responses to long‐term, arduous military training

**DOI:** 10.14814/phy2.70915

**Published:** 2026-05-19

**Authors:** Tyler E. Oliver, Anna V. Oppenheimer, Emily S. Lange, Samantha J. Goldenstein, P. Matthew Bartlett, Jess A. Gwin, Kristin J. Heaton, Holly L. McClung

**Affiliations:** ^1^ U.S. Army Research Institute of Environmental Medicine (USARIEM) Natick Massachusetts USA; ^2^ Oak Ridge Institute for Science and Education (ORISE) Oak Ridge Tennessee USA

**Keywords:** army ranger, female, military, perseverance, resilience, women

## Abstract

The U.S. Army Ranger Training Course (RTC) challenges candidates physically and mentally over 61+ days across varied environments. Psychological measures of resilience, grit, sleep, and physical performance have been investigated during RTC; however, whether those associations differ between sexes has not, despite sex integration occurring in 2015. 37 participants (10F, 27 ± 3y; 27 M, 25 ± 4y; mean ± SD) completed measurements of strength and power (isometric mid‐thigh pull (IP) and standing long jump (SLJ)), self‐reported outcomes of sleep (Pittsburgh Sleep Quality Index (PSQI)), resilience (CD‐RISC‐10), and grit (GRIT‐S) before (BL) and after RTC (POST). Males displayed greater absolute measures of IP and SLJ at BL and POST than females (both, *p* < 0.0001); however, both sexes showed similar deficits POST. Sleep declined over time, with PSQI scores increasing (*p* < 0.001), including daytime dysfunction, duration, and quality deteriorating similarly for both sexes (all *p* < 0.05). GRIT‐S and CD‐RISC10 scores started and remained high, with no change over time nor differences by sex. Large deficits in strength and sleep were observed in a mixed‐sex sample of military personnel undergoing strenuous 61+ day elite military training. Although males displayed greater absolute strength and power than females at both time points, no differences in performance decrements, sleep, or psychological factors were observed over the course.

## 1 INTRODUCTION

Occupational stress is ubiquitous and can influence workers' health and overall well‐being (Bolger et al., 1989; Charles et al., 2013; Kivimäki et al., 2002; Piazza et al., 2013). Many stressors, such as difficult interpersonal relationships and changing organizational demands and priorities, are common to most job environments. However, tactical populations (i.e., military, law enforcement, firefighter, and rescue personnel) experience a unique combination of physical and mental demands during training and operations that require continuous adaptation and high levels of preparedness (Scofield & Kardouni, 2015; Xu et al., 2023). Within the modern military operational environment, success depends on proficient physical and mental performance.

Historically, research examining performance in military contexts has focused predominantly on physical factors (Harty et al., 2022; Nindl et al., 2002; Winters et al., 2021). More recently, the impact of non‐physical factors, such as psychological resilience, grit, and sleep on military servicemembers' performance has received more attention. For example, a significant body of evidence indicates that sleep loss is associated with impairments in attention (Hudson et al., 2020) and cognitive function, including judgment and decision‐making (Heaton et al., 2022; Killgore et al., 2006; Smith et al., 2019; Womack et al., 2013), all of which are critical components of effective task performance. In addition, high levels of measured psychological resilience (ability to adapt and grow in positive ways through difficult or stressful experiences (Bezdjian et al., 2017; Ledford et al., 2020)) and grit (passion and perseverance to achieve long term goals (Duckworth et al., 2007)) have each been associated with successful completion of military training for both male and female Soldiers (Kelly et al., 2014; Maddi et al., 2017). Moreover, higher self‐rated psychological resilience has been shown to predict lower levels of perceived stress and mental distress, and better observer‐rated military performance in military trainees (Sefidan et al., 2021). Such traits are essential for managing the prolonged stress of not only training, but also future combat and deployment exposure.

To date some work in the military environment has been done with comparatively fewer studies conducted in elite combat training courses like the U.S. Army Ranger Training Course (RTC) or Special Forces Assessment and Selection (SFAS). These training courses serve as unique and controlled environments to assess individual Soldier responses to the extreme stressors of prolonged physical activity (16‐22 h/d;~4100 kcal/d) coupled with food (2500–4500 kcal/d) and sleep restriction (3–4 h/night), all of which characterize typical operational conditions (Moore et al., 1994; Nindl et al., 2007; Shippee et al., 1995). RTC is a 61+ day, multi‐phased course designed to assess individual Soldier tactical and leadership skills under extreme mental and physical stress. The RTC consists of three unique phases of military training, each lasting ~20 days, each under different environmental conditions starting with a basic field phase (Camp Darby, Fort Benning, GA); followed by a mountains phase (Camp Merrill, Dahlonega, GA), and final swamp phase (Camp Rudder, Eglin Air Force Base, FL). RTC incorporates intentional sleep restriction and a high‐stress environment to provide a realistic assessment of a Soldier's ability to cope with challenges and successfully execute mission tasks in a combat scenario. Prior RTC research, before full integration of sexes, was exclusive to male candidates documenting significant drops in total body mass (−16%) (Nindl et al., 2007), elevations in serum cortisol (Friedl et al., 1994) and average daily sleep of 3.2–3.6 h per night with some individuals reporting less than 2 h per night, depending on course phase (Pleban et al., 1990; Shippee et al., 1995).

To date, only limited research during military operations has included females or sex differences in outcomes, largely due to the exclusion of U.S. servicewomen from combat prior to 2015. Available studies focused on short‐term field training exercises with limited stressors, examining differences in body composition, energy expenditure, and physical performance reporting limited differences across sex (Castellani et al., 2006; Hoyt et al., 2006; Vikmoen et al., 2020). Hoyt et al. (2006) observed absolute differences in total energy expenditure (TEE) and fat‐free mass (FFM) loss between male and female Norwegian cadets across a 7‐day FTX, however sex differences were no longer evident when standardized to body mass (Hoyt et al., 2006). Castellani et al. (2006) examined male and female Marine recruits during a 54‐h FTX with TEE and FFM showing no sex differences when normalized to body mass (Castellani et al., 2006). Lastly, Vikmoen et al. (2020) observed similar performance declines with no difference across male and female Norwegian soldiers during a 5.5 days FTX with countermovement jump, medicine ball throws, and anaerobic test scores (Vikmoen et al., 2020). More recently, research within RTC cohorts focused on characterizing psycho‐social traits of females graduates from elite military combat training courses (e.g., RTC and Marine Infantry Officer Course) in which they displayed high levels of grit and resilience (Tharion et al., 2023), similar to male Soldiers going through SFAS courses. Among a male‐only population of elite Soldiers, higher physical performance, grit, and resilience scores were all predictive of selection into SFAS over a 20‐day training period (Farina et al., 2019). However, the question remains whether males and females respond similarly to such high stress and performance environments, and to what extent physical and non‐physical factors such as altered sleep patterns occur throughout strenuous, prolonged military training.

The aim of this study was to assess, characterize, and compare the physical performance and non‐physical responses (grit, resilience, and sleep outcomes) of male and female Soldiers completing 61+ days of rigorous military training with a multitude of stressors. We hypothesized that male and female Soldiers would experience similar declines in measures of strength (isometric mid‐thigh pull), power (standing long jump), sleep, and report similar levels of grit and resilience throughout prolonged simulated military operations.

## 2 METHODS

This was a sub‐study of a larger parent study focused on sex differences in energy balance and body composition changes during the RTC (McClung et al., 2026). This study was approved by the Human Use Review Committee at the U.S. Army Medical Research and Development Command Human Institutional Review Board (Ft. Detrick, MD) and all tests and protocols were in accordance with the *Declaration of Helsinki*. Investigators adhered to the policies regarding the protection of human subjects as prescribed in DODI 3216.02 and the research was conducted in adherence with the provisions of 32 CFR part 219.

### 2.1 Course description and participant recruitment

All study participants successfully completed RTC. To pass each of the three RTC phases candidates were challenged to demonstrate proficiency in tactical planning, communication, leadership, and resource allocation to execute successful operations during multiple field training exercises (FTXs). Phase success was determined by evaluations from both course instructors and peers. Receiving positive evaluations allowed a candidate to move on to the next RTC phase. RTC leadership maintain standards across the training equally for every RTC candidate, regardless of sex. All candidates (therefore study participants) had the same course standards, requirements and conditions. There were no differences in the equipment issued, rucksack load carried, ration amount or type distribution, rest‐work cycles, etc. across candidates. Loads and equipment varied equally across candidates throughout the course and phases based on individual roles (e.g., leadership vs. non‐leadership; patrols, etc.). Candidates that failed an evaluation (peer or instructor) at each phase were either ‘recycled’ (repeated the failed phase) or ‘failed’ RTC (released from the course). Each candidate is allowed up to two ‘recycles’ per phase before failing out of RTC. Failure of a phase is common; on average successful RTC candidates ‘recycle’ at least one phase (Moore et al., 1994; Nindl et al., 2007; Shippee et al., 1995). Commonly the duration of RTC is cited as ‘61 + d’ to account for additional time in course required by phase ‘recycling.’

Study recruitment was rolling with participants recruited from six RTC classes from April–September 2023 and data collected from April 2023 to January 2024. Participants provided written informed consent during enrollment at RTC following the study briefing on purpose and risks. Participants were 18 years or older, and current U.S. military servicemembers in active duty, reserve or National Guard. In total, 37 participants (10 female, 27 male) completed all phases of the RTC course (“course completers”). Baseline characteristics of these 37 course completers are described in Table 1.

**TABLE 1:** Baseline characteristics of female and male participants.

	Females (*n* = 10)		Males (*n* = 27)	
	Mean ± SD	Min – Max	Mean ± SD	Min–max
Age (y)	27 ± 3	23–32	25 ± 4	19–34
Anthropometrics				
Height (cm)	167.0 ± 5.8	156.0–174.0	177.0 ± 8.7	164.0–194.0
BM (kg)	69.3 ± 6.5	62.9–85.1	82.7 ± 9.0	65.0–98.8
BMI (kg/m^2^)	24.8 ± 1.8	22.6–28.3	26.3 ± 2.1	23.4–32.7
Race/Ethnicity				
White	8 (80%)		21 (77.8%)	
Black	‐		1 (3.7%)	
Hispanic	‐		2 (7.4%)	
Asian	2 (20%)		2 (7.4%)	
Missing	‐		1 (3.7%)	
Education Level (%)				
High school diploma	1 (10%)		5 (18.5%)	
Some college	‐		6 (22.2%)	
Associate's degree	‐		2 (7.4%)	
Bachelor's degree	7 (70%)		10 (37.0%)	
Graduate courses/degree	2 (20%)		3 (11.1%)	
Missing	‐		1 (3.7%)	
Rank				
Junior enlisted	0 (0%)		13 (48.1%)	
Senior enlisted	1 (10%)		3 (11.1%)	
Junior Officer	9 (90%)		11 (40.7%)	

*Note*: Data are presented as mean ± standard deviation (SD) and minimum – maximum.

Abbreviations: BM, body mass; BMI, body mass index.

### 2.2 Study design

**FIGURE 1:**
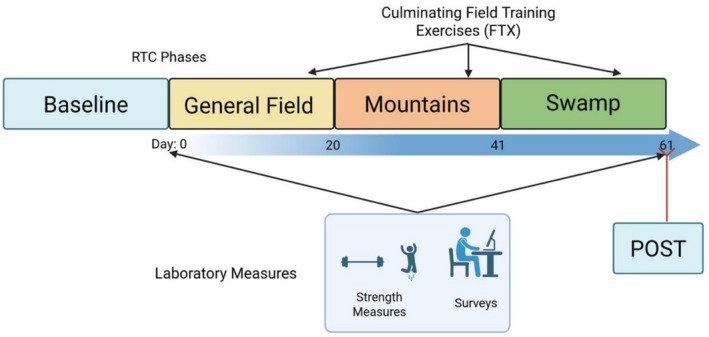
Timeline of ranger training course. FTX, field training exercise; POST, post‐RTC, ranger training course.

Data collection took place prior to the RTC course start (baseline, BL) and following completion (POST) (Figure 1). Baseline measurements of physical performance, psychological factors, and sleep were taken on day 0 of the course at approximately 1500–1900 h. POST data collection mirrored BL and immediately followed the culminating event of the last field training exercise (approximately RTC Day 56, from 2200 to 0300 h). Study participants that recycled a RTC phase were able to maintain normal sleep and feeding practices until they reinserted into a phase (on average ~20 days).

### 2.3 Anthropometric measurements

Baseline measurements of height and body mass (BM) were taken in minimal uniform clothing (e.g., t‐shirt, pants, and socks) with boots removed. Standing height was measured to the nearest 0.1 cm and BM with to nearest 0.1 kg with an electronic scale (Seca 769). Body mass index (BMI) was calculated and utilized to provide an assessment of body size.

### 2.4 Physical strength and power

Following a standardized dynamic warm‐up (e.g., squats, lunges, progressive squat jumps, etc.) trained study staff followed standard operating procedures to assess participants' power and strength with the standing long jump (SLJ) and isometric mid‐thigh pull (IP), respectively, to assess physical performance at each timepoint.

#### 2.4.1 Standing long jump

The SLJ test was performed using a standardized rubber mat (Kinesiology Book Publisher, Toronto, Canada) with markings to measure jump distance. Participants were instructed to stand behind a marked line with feet shoulder‐width apart; a two‐foot take‐off with arm swinging was allowed to provide forward drive. Participants were instructed to jump as far as possible and ‘stick’ their landing on the mat to allow measurement post‐jump. Measurements of distance were taken at the back of the participant's feet. Each participant was allowed three jumps with ~30 s of rest between attempts. Participant would repeat a jump if they fell backwards or moved their feet prior to marking a measurement marked. An overall power score was quantified as the average of the two furthest jumps and used for data analysis (Koch et al., 2003).

#### 2.4.2 Isometric mid‐thigh pull

A back and leg dynamometer (Takei Scientific Instruments Co. Ltd., Niigata, Japan) was used to assess participants' lower‐body (e.g. back, leg) maximal isometric strength for the IP test. Following visual demonstration, participants stood on a footplate with their knees flexed to ~115° and hips to 30°, verified using a manual goniometer. Participants were instructed to use an overhand grip on the pull‐bar attached to the dynamometer and pull the bar in a vertical direction with maximal effort by attempting to extend through the hips and knees while also maintaining the stable initial starting position (Dawes et al., 2017; Nickels et al., 2020). A trained study team member manually recorded the highest observed value held for 3 s for each attempt. Three attempts were given with ~30–60 s of rest between attempts with the maximal attempt used for data analysis.

### 2.5 Psychological and sleep outcomes

Surveys were distributed electronically via Survey Tracker software (Scantron, Eagan, MN) and responses were used to assess self‐reported grit, resilience, and sleep metrics at both timepoints.

#### 2.5.1 Grit

The short grit scale (GRIT‐S) is a modified and validated version of the original GRIT scale with eight questions assessing perseverance and passion for long‐term goals (Duckworth et al., 2007; Duckworth & Quinn, 2009). Respondents used a 5‐point Likert scale ranging from 1 (“not like me at all”) to 5 (“very much like me”) to rate how much each statement accurately reflects how they feel. An overall score was obtained by averaging ratings, with higher scores reflecting higher levels of each grit component.

#### 2.5.2 Resilience

The Connor‐Davidson Resilience Scale (CD‐RISC‐10) (Campbell‐Sills & Stein, 2007) is a validated survey that uses a 5‐point scale from zero (“not true at all”) to four (“true nearly all the time”) to indicate the extent to which respondents believe each statement reflects how they feel (Campbell‐Sills et al., 2006; Connor & Davidson, 2003). A total score was obtained by summing the 10 items; total scores range from 0 to 40, with higher scores reflecting greater levels of resiliency (Campbell‐Sills & Stein, 2007).

#### 2.5.3 Pittsburgh sleep quality index (PSQI)

The PSQI is a validated, 19‐item self‐report questionnaire of sleep quality and disturbances in adults (Buysse et al., 1989). Respondents are asked to rate the extent to which each item reflects their typical sleep habit during the preceding month. Thus, BL PSQI values reflect participants' typical sleep habits in the month before the start of training, and the POST PSQI reflects participants' self‐rated sleep habits during the 30 days prior to completion of training. Responses are grouped across 7 component factors: Sleep Quality (Component 1), Sleep Latency (Component 2), Sleep Duration (Component 3), Sleep Efficiency (Component 4), Sleep Disturbances (Component 5), Sleep Medication Use (Component 6), and Daytime Dysfunction (Component 7). Each component score is weighted equally, with scores ranging from 0 to 3 (higher scores indicating poorer sleep quality). A Global PSQI measure, with values ranging from 0 to 21, is the sum of the 7 component scores and is considered to be an indicator of overall sleep quality. Global scores greater than 5 have been used to distinguish poor sleepers (Buysse et al., 1989).

### 2.6 Statistical analysis

Although performance (physical and non‐physical) outcomes were not the main hypothesis used for sample size calculations conducted a priori, the study was powered to observe effects on outcomes reported herein. Sample size to detect within group change was estimated using mean change and SD in key outcomes of the main study (e.g., body composition and energy balance) based on findings of similar research (Hoyt et al., 2006). With consideration of the lack of existing data in females, a simulation using various standardized E/S (medium = 0.5, large = 1.0, and very large = 2.0) was used to detect longitudinal changes within and between sex with E/S sizes ≥1.0 (large). With application of a 10% continuity correction for samples sizes <30, it was estimated the total sample size required to detect longitudinal changes within groups was *n* = 8 study participants completing RTC. Therefore, the final sample size (*n* = 37) reported herein (F, *n* = 10 and M, *n* = 27) was sufficient to detect overall longitudinal changes within groups and differences in longitudinal changes between sex.

Only study participants that completed RTC were included in the final analysis. For participants that recycled a phase, only measures collected at initial BL and during the final successful POST phase attempt were selected for analyses. To first assess the associations of sex with each of the primary outcomes of interest (GRIT‐S, CD‐RISC‐10, PSQI, IP, and SLJ), multivariable linear regression models, adjusted for baseline age as a covariate, were used.

Linear mixed models (LMM), with timepoint, BL age, and sex set as fixed effects and participant as a random effect, were then used to examine the differences in each variable of interest from BL to the end of RTC (POST). An interaction term between timepoint/phase and sex was subsequently included to evaluate if differences in outcomes over time differed by sex. Additional LMMs included PSQI global scores as a fixed effect to assess its impact on IP and SLJ measurements over time. Subsequent models included an interaction between PSQI global scores to evaluate if differences in IP and SLJ over time differed by sex. Statistical significance of the interaction term effect estimate set at the *p* < 0.05 level, as well as a likelihood ratio test was used to compare nested models with and without the interaction term were used to determine whether sex differences were statistically significant. Sex‐stratified LMMs with timepoint and baseline age set as fixed effects and participant set as a random effect were employed to examine differences in each variable over time separately among males and females. Missing data refers to a participant's inability (i.e., due to injury, unavailable due to course requirements) to complete a measurement at a specific timepoint for the IP (1/74 observations; ~1% data) and the SLJ (6/74 observations; ~8% data). To account for missing data, multiple imputation by chained equations was performed with 10 imputed datasets, using the mice package in R (van Buuren & Groothuis‐Oudshoorn, 2011). Statistical significance was set at *p* < 0.05. All analyses were conducted using R version 4.4.1 (R Core Team, Vienna, Austria).

## 3 RESULTS

At BL, female and male participants were of similar age (mean ± SD: 27 ± 3 and 25 ± 4 years, respectively; Table 1). Male participants were heavier and larger compared to female counterparts with anthropometric measures of height (177.0 ± 8.7 vs. 167.0 ± 5.8 cm, respectively; *p* < 0.001), and measures of body mass (82.7 ± 9.0 vs. 69.3 ± 6.5 cm; *p* < 0.001), and body mass index (*p* < 0.05) differing by sex. Female participants were predominantly junior officers (90%) and had high levels of education (90% bachelor's degree or higher). Male participants were largely enlisted personnel (~60%) and varying degrees of education. Mean time in RTC did not differ by sex for the 37 course completers (*p* = 0.99). Seventeen (4F, 13 M) went straight through RTC in 61 days while the remaining 20 recycled at least one phase (6F, 14 M) with an average course duration of 94 ± 15 days.

### 3.1 Physical strength and power

**FIGURE 2:**
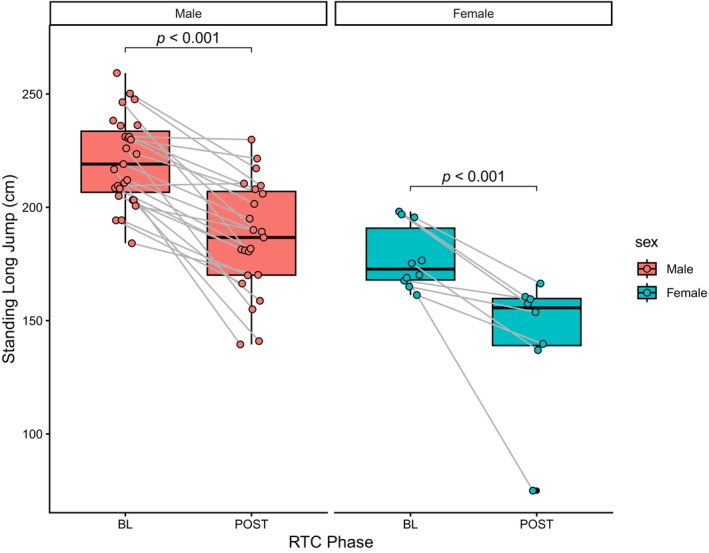
Standing long jump across ranger training course. BL, baseline; POST, post‐RTC; RTC, ranger training course; individual data represented at BL (*n* = 27 M; 10 F) and POST (*n* = 23 M; 8 F). There were absolute differences across sex at BL and POST. Both male and female participants displayed significant decreases in SLJ over time. Data were compared using linear mixed models (LMMs) adjusted for baseline age. The solid line in the box represents the median, with hinges of the box (i.e., top and bottom lines) representing the 75th (top) and 25th percentile (bottom). The whiskers are data points that lie at the maximum (top) and minimum values (bottom). Black dots indicate outliers as defined by 1.5x the interquartile range for Q1 and Q3. Some data points may overlap.

There was a significant overall decrease in SLJ distance from BL (mean ± SD: 209 ± 26 cm) to POST (175 ± 32 cm; Estimate [95% Confidence Interval] −29.5 cm [−39.6, −19.5], *p* < 0.001). A main effect of sex was observed in the LMM (−56.2 cm [−88.8, −23.7], *p* = 0.001), confirmed by within phase comparisons showing an absolute difference in males compared to female counterparts with SLJ distance at BL (221 ± 19 vs. 178 ± 14 cm, respectively; *p* < 0.0001) and POST (187 ± 25 vs. 144 ± 30 cm, respectively, *p* < 0.0001). However, the decrement in SLJ over time from BL to POST (males: −28.3 cm [−38.1, −18.5], *p* < 0.001; females: −33.6 cm [−54.7, −12.5], *p* = 0.005) did not differ between sex (*p* = 0.8; Figure 2). Sleep (PSQI global score) impacted SLJ distance in the overall group (−2.44 cm [−4.7, −0.2], *p* = 0.034); there was no interaction between PSQI global score and sex on the change in SLJ from BL to POST (*p* = 0.493).

**FIGURE 3:**
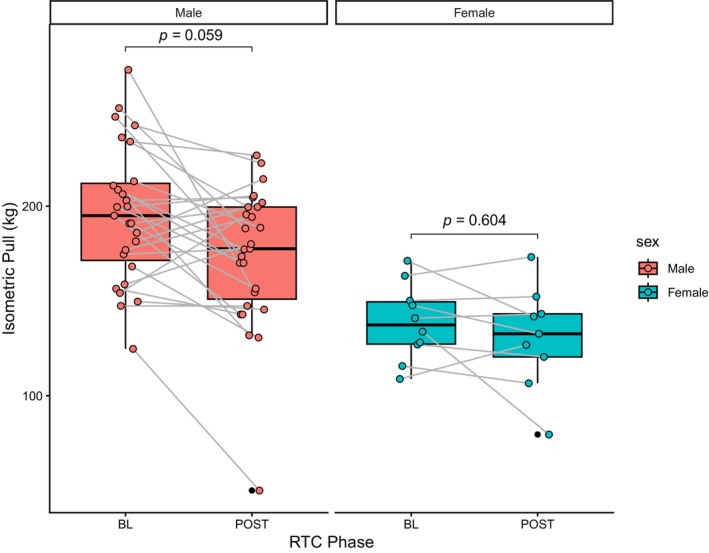
Isometric pull across ranger training course. BL, baseline; POST, post‐RTC; RTC, ranger training course; individual data represented at BL (*n* = 27 M; 10 F) and POST (*n* = 27 M; 9 F). There were absolute differences across sex at BL and POST. Neither male nor female participants displayed statistically significant change over time. Data were compared using linear mixed models (LMMs) adjusted for baseline age. The solid line in the box represents the median with hinges of the box (i.e., top and bottom lines) representing the 75th (top) and 25th percentile (bottom). The whiskers are data points that lie at the maximum (top) and minimum values (bottom). Black dots indicate outliers as defined by 1.5× the interquartile range for Q1 and Q3. Some data points may overlap.

Similarly, changes in physical strength as measured by IP overall were significant over time (−24.3 kg [−40.2, −8.4], *p* = 0.003) and by sex (−58.9 kg [−84.0, −33.7], *p* < 0.001), however the magnitude of change between BL and POST did not differ by sex (*p* = 0.263; Figure 3). Although a sex difference was detected between IP strength at BL (196 ± 36.1 vs. 139 ± 19.9 kg, respectively; *p* < 0.0001) and POST (174 ± 36.9 vs. 131 ± 27.1 kg, respectively; *p* < 0.0001). No effect of sleep scores on the change in IP was detected, nor was an interaction between sleep and sex on IP outcomes observed (*p* > 0.05 for both).

### 3.2 Sleep

Overall, self‐reported sleep outcomes deteriorated from BL to POST, with PSQI global scores increasing from 6.60 ± 2.74 at BL to 8.97 ± 1.98 at POST (*p* < 0.001). Sex‐stratified results (Table 2) show an increase in PSQI scores for males (2.6 pts. [1.5, 3.4], *p* < 0.001) but not for females (estimate: 1.8 pts. [−0.4, 4.0], *p* = 0.09) across time. Male and female Soldiers displayed similar changes in all seven PSQI component scores across time with daytime dysfunction (both *p* < 0.05), sleep duration (both *p* < 0.001), and subjective sleep quality (both *p* < 0.05) decrements from BL to POST. Component scores for sleep latency decreased for both sexes, indicating that participants reported falling asleep more quickly (*p* < 0.01). No changes over time (BL to POST) were experienced for either males or females for sleep medication use, sleep disturbance, and sleep efficiency component scores (all, *p* > 0.05). No sex difference was detected across all seven PSQI component scores in the magnitude of change from BL to POST (all, *p* > 0.05) (Table 2).

**TABLE 2:** Change in PSQI global and component scores from BL to POST.

PSQI component	Mean score ± SD				Sex interaction (*p*‐value)
	Females (*n* = 10)		Males (*n* = 27)		
	BL	POST	BL	POST	
Sleep duration	0.90 ± 1.20	3.00 ± 0***	1.42 ± 1.03	3.00 ± 0***	0.19
Sleep latency	1.10 ± 0.57	0.22 ± 0.67**	1.12 ± 0.95	0.26 ± 0.66***	0.99
Sleep quality	1.30 ± 0.48	2.00 ± 1.00*	1.44 ± 0.71	2.33 ± 0.96***	0.48
Sleep efficiency	0.60 ± 0.84	0.22 ± 0.67	0.62 ± 0.75	0.74 ± 1.02	0.14
Sleep disturbances	1.10 ± 0.57	0.78 ± 0.67	1.08 ± 0.48	0.89 ± 0.58	0.55
Sleep medication use	0.50 ± 1.08	0.11 ± 0.33	0.35 ± 0.80	0.15 ± 0.60	0.60
Daytime dysfunction	0.90 ± 0.88	1.89 ± 1.05*	0.77 ± 0.77	1.85 ± 0.91***	0.74
PSQI global score	6.40 ± 2.67	8.22 ± 1.86	6.68 ± 2.81	9.22 ± 1.99***	0.45

*Note*: Each PSQI component is weighted to a max of 3 points. Data are presented as mean (±SD). *p < 0.05, **p < 0.01, ***p < 0.001 indicates difference from BL.

Abbreviations: BL, baseline; POST, Post‐RTC, Ranger Training Course; PSQI, Pittsburgh Sleep Quality Index.

### 3.3 Grit and resilience

Self‐reported outcomes of grit and resilience did not differ by sex at either timepoint (Table 3). GRIT‐S scores showed no change from BL (3.85 ± 0.45 M, 4.00 ± 0.28 F; *p* = 0.3) to course completion (3.84 ± 0.45 M, 4.06 ± 0.33 F; *p* = 0.3). Similarly, CD‐RISC‐10 scores did not differ across sex or by time (BL: 32.7 ± 3.78 M vs. 30.4 ± 4.50 F, *p* = 0.2; POST: 32.1 ± 4.94 M vs. 29.4 ± 3.19 F; *p* = 0.2). Models found no change in the magnitude of change across sex from BL to POST in mean GRIT‐S (*p* = 0.8) and CD‐RISC‐10 (*p* = 0.2) scores for RTC participants.

**TABLE 3:** Change in GRIT‐S and CD‐RISC‐10 Scores from BL to POST.

		Females (*n* = 10)		Males (*n* = 27)		Sex interaction (*p*‐value)
		Mean ± SD	Δ (95% CI)	Mean ± SD	Δ (95% CI)	
GRIT‐S	BL	4.00 ± 0.28	‐	3.85 ± 0.45	‐	0.3
	POST	4.06 ± 0.33	0.1 (−0.2, 0.3)	3.84 ± 0.45	0.0 (−0.1, 0.1)	0.3
CD‐RISC‐10	BL	30.4 ± 4.50	‐	32.7 ± 3.78	‐	0.2
	POST	29.4 ± 3.19	−0.8 (−3.7, 2.1)	32.1 ± 4.94	−0.9 (−2.2, 0.5)	0.2

*Note*: Data are presented as mean (±SD). Differences as 95% CI. Δ is difference from baseline.

Abbreviations: BL, baseline; CD‐RISC‐10, Connor‐Davidson Resilience Scale; GRIT‐S, short grit scale; POST, post‐RTC, ranger training course.

## 4 DISCUSSION

This study is the first to assess both physical and non‐physical performance factors including strength, power, grit, resilience, and sleep in a mixed‐sex sample of military personnel undergoing prolonged, strenuous elite military training. Absolute measures of IP and SLJ were greater for males than females both at the start (BL) and completion (POST) of RTC; however, interestingly, both sexes experienced a similar magnitude of decline in these strength and power assessments over the course. Similarly, the degree of change in measures of psychological resilience, grit, and sleep across the course (BL to POST) was not different by sex. These results document that Soldiers, male or female, experienced similar responses and decrements when exposed to identical physical, mental, and environmental stressors of the RTC. Thus, the lack of sex differences reported herein provides novel additions to understanding the holistic response of an integrated Force completing this modern (integrated) elite operational training.

Significant declines in physical performance measures of lower body power and strength were observed for all Soldiers. SLJ displayed a large decrease for both males (−34 cm; −15.4%) and females (−34 cm; −19.5%) over the 61+ days of military training. These declines were larger than observed in previous work (~6.5%) with male Soldiers capturing performance outcomes of similar types of military field training, but shorter duration (7–21 days) (Margolis et al., 2014; Ojanen et al., 2018; Ritland et al., 2021; Szivak et al., 2018; Welsh et al., 2008). Outcomes reported herein were comparable to earlier studies of all male RTC participants where a 16% decline in vertical jump height was observed by Nindl et al. (Nindl et al., 2007) following U.S. Army RTC, and a decline of more than 13% was found in SLJ performance by Singaporean Army Ranger candidates during training of a similar duration (Gan et al., 2022). Maximal isometric mid‐thigh pull strength decreased 11% for males and 6% for females from BL to POST in the current study, reflecting smaller declines than the ~20% decline in maximal lifting strength and power output reported by Nindl et al. (Nindl et al., 2007) in male participants before and after RTC. The variation in the degree of strength decline relative to prior studies may be due in part to methodological differences in the assessment of performance (e.g., vertical jump height vs. maximal lift strength) or related to (RTC) course modernization in the 30+ years lapse from when earlier studies (Moore et al., 1994; Shippee et al., 1995) were completed. For example, current U.S. Army RTC standard issue is two complete Meal‐Ready‐to‐Eat (MRE™) rations, whereas in 1992, just one MRE ration was issued (~1300 kcal/d vs. modern issue ~2700 kcal/d) perhaps minimizing caloric deficit thereby impacting physical performance.

The physiological basis for sex differences in physical performance has been a topic of recent interest (Hunter et al., 2023; Hunter & Senefeld, 2024). One hypothesis for the sex gap in performance stems from male exposure to endogenous testosterone (Joyner et al., 2024; Senefeld et al., 2020), influencing muscle fiber morphology (Staron et al., 2000) and ultimately power‐based performance (Senefeld et al., 2013). Findings reported herein illustrate similar outcomes with males displaying greater absolute strength compared to females both at BL and POST, however despite greater strength and power in male Soldiers, the change in performance decrement across the course did not differ by sex. In the context of physical demands in military occupational specialties (MOS) or jobs, an MOS defined in a ‘heavy physical demand’ category (e.g., RTC tasks and most course graduates), include requirements for frequent physical tactical tasks such as “occasionally lifting a 238 lb [108 kg] individual a distance of 3 ft as a member of a two Soldier team (assumed prorated at 119 lb [54 kg] per Soldier)” and “frequent raising, carrying, or lifting of 110 lb [50 kg] a distance ~1.5 m on a pivot point” (Regulation A 611‐21, n.d.). Female strength reported herein, post‐RTC with noted degradation, would still allow them to successfully complete required ‘heavy’ demand tasks as described with mean female IP strength of 131 kg (~289 lb). These findings indicate females are as capable and physically‐resilient as males following prolonged training in an austere environment.

Both male and female participants entered the RTC with relatively high global PSQI scores, classifying them as “poor” sleepers per standardized cut‐off scores established by Buysse et al. (Buysse et al., 1989). BL values reported in this study are similar to previous work describing outcomes from U.S. Army Rangers (Ritland et al., 2023) and active‐duty Soldiers (Ritland et al., 2024). Regardless of sex, Soldiers entered RTC with similar sleep patterns and exhibited similar deterioration of self‐reported values of sleep duration, subjective sleep quality, and daytime dysfunction (higher PSQI component scores) throughout the course (BL to POST). Sleep latency declined (lower component score) from BL to POST for both males and females, reflecting increased sleep pressure. Notably, these POST values are comparable to active‐duty military personnel presenting with clinical sleep disorders (insomnia, sleep apnea) and service‐related illness (Matsangas & Mysliwiec, 2018). The large decreases in sleep quality and dysfunction are likely due to the inherent design of the RTC to impose high levels of physical activity with purposeful caloric deprivation and sleep restriction aimed to simulate operational scenarios. Although programmed and purposeful, loss of sleep and resulting fatigue can impair physical and cognitive performance, including deficits in learning and execution of complex military skills such as rifle marksmanship (McLellan et al., 2005; Smith et al., 2019; Tharion et al., 2003). Considering that sleep restriction is an intentional component of this military training, disruptive factors during training contributing to poor sleep quality (i.e., noise, light) were not controlled for in this study, reflecting real‐world military operational contexts. The lack of sex differences in sleep outcomes reported herein aims to addresses the gap in research to explore the effect of long‐duration training and the consequences of heavy physiological and mental demands over time.

The inherent impact of chronic sleep loss on outcomes of physical strength and power measures was of particular interest due to evidence of degraded performance in athletic populations following acute periods of sleep restriction (Rae et al., 2017; Roberts et al., 2019). Our findings were mixed when assessing the impact of self‐reported sleep outcomes by PSQI global scores. The PSQI scores had an overall negative effect on SLJ distance with no impact related to IP strength. The lack of consistency in our findings may relate to the study's small sample size and require further research in a larger population. Provided the magnitude of sleep decline reported in both male and female participants, future research should place emphasis on recovery from prolonged exposure to multi‐stressor environments, such as RTC. This has particular application to the civilian athletic population with recommended guidelines for relative energy deficiency in sport (RED‐S) (Mountjoy et al., 2023) with links to longer‐term health outcomes, such as the relationship between sleep deficits and musculoskeletal injuries (Ritland et al., 2023; Ritland et al., 2024).

To date, this is one of the first studies to examine sex differences in, and moderators of, grit and its relationship to overall performance of physical strength and power during long‐term military training. As previously mentioned, training schedule constraints limited the amount of time available for assessment. The selection of these specific measures of grit and resilience was driven in part by evidence in the literature suggesting their relevance to training outcomes (Eskreis‐Winkler et al., 2014; Farina et al., 2019; Grier et al., 2020; Killgore et al., 2006; Lovering et al., 2015) and by their inclusion in earlier studies conducted by our team in elite military training cohorts, thus permitting cross‐study comparisons. As might be expected in a cohort of high‐achieving military personnel completing a highly competitive military leadership training, males and females reported similarly high scores at both BL and POST. Mean grit scores reported herein align with those of previous studies of elite military training courses (Benedict et al., 2023; Eskreis‐Winkler et al., 2014; Tharion et al., 2023) but are higher than scores reported by individuals attending military leadership academies (Duckworth et al., 2007) and initial military training (Duckworth et al., 2019; Kelly et al., 2014). While the RTC is physically taxing, the mental burden placed on the participants during periods of leadership evaluation over 61+ days requires long‐term perseverance to complete the course. Since, at the time of POST data collection, participants had not been notified if they had successfully graduated from the course, the findings reported here could indicate a ceiling effect, although we cannot rule out the potential influence of social desirability bias (Duckworth et al., 2007).

Similar to grit, self‐reported psychological resilience across RTC did not differ over time or by sex. Mean CD‐RISC‐10 scores reported are consistent with those of elite athletes and healthy U.S. adults (30.6 and 31.4, respectively) (Gonzalez et al., 2016; Paulus et al., 2012) as well as populations with frequent occupational stress, such as trauma surgeons (33.4) (Warren et al., 2013) and elevated relative to populations such as college undergraduates (27.2), medical students (28.2), and nurses (30.7) (Campbell‐Sills & Stein, 2007). Evidence to date supports the existence of higher psychological resilience as a potentially protective factor against operational stress such as sleep‐loss and increased cognitive load (Hughes et al., 2018; Mantua et al., 2021). Within the military, attention has been focused on methods aimed at improving Soldier resilience to facilitate stress management and coping strategies during periods of adversity (Hernandez et al., 2024; Hernandez et al., 2025; Zueger et al., 2023) and improve retention (Knapik et al., 2004; Navy, 2016). The positive benefits of such training have been reported in military populations (Adler et al., 2011; Cohn & Pakenham, 2008; Foran et al., 2012) and in civilian cohorts (Chesak et al., 2019; Chesak et al., 2021; Loprinzi et al., 2011; Seshadri et al., 2020; Sood et al., 2014). Further investigation of the impact of resilience training on preparation for and performance during arduous military training courses, specifically those emphasizing leadership skills, such as RTC, would be beneficial. Evidence from this study suggests male and females who successfully complete arduous military training are equally gritty and resilient, and maintain their grittiness and resilience comparably, in response to stressful physical and mental‐loads over long‐durations. The lack of sex differences reported in this study highlight the importance in considering psychosocial traits in combination with physical strength and power to provide a more holistic understanding of training adaptations in both male and female athletes.

## 5 LIMITATIONS

The findings from this study are limited by the small sample size. This is inherent to the high attrition that occurs throughout the RTC, particularly in the initial phase which entails an intensive 5‐day physical performance assessment resulting in ~50% failure rate. Additionally, very few females enroll in the RTC with only ~175 graduates [personal communication, Kris Fuhr, 2026] since combat military occupational series opened to females in 2015. Further, due to the relatively small sample size, analyses comparing RTC completers to non‐completers such as identifying predictors of RTC completion were not feasible. Further, given that females make up a small proportion of RTC completers, males were oversampled to improve the overall sample size and power. Although minimal data was missing, in cases where data points were missing, multiple imputation methods were used to account for missingness. Other limitations include the use of self‐report measures of sleep quality and duration (PSQI), grit (Grit‐S), and resilience (CD‐RISC‐10). Responses to subjective measures such as these can be influenced by factors such as motivation and social desirability. In this competitive military training environment, participants may have over‐reported positive traits and behaviors based on perceived expectations and the evaluative nature of the training environment. To address the potential impact of bias in self‐reporting, participants were encouraged to respond as honestly and accurately as possible and were informed that any results would not be shared with RTC instructors and results would be presented as aggregate and not individual data. The absence of non‐completers from the analytic sample limits potential comparisons that could shed light on potential desirability biases. Working within the confines of the field study, with limited access and footprint (e.g., minimize impact on the live military training) the assessment of performance was limited to low risk and short duration strength and power testing. It was not feasible to accurately assess aerobic performance as part of this research. Although effort was made to adhere to a consistent schedule for data collections across training classes (e.g., same day of course, same time of day), access to participants was contingent on completion of training activities, which varied from class to class. Variability in timing of assessments could impact measures of physical strength and power, as well as self‐appraisals of psychological states (Facer‐Childs et al., 2018; Reilly et al., 1997). Finally, due to the nature of the study and limited number of female RTC candidates it was not possible to control for menstrual cycle across female study participants, therefore links to menstrual phase and performance outcomes could not be controlled for in this study.

## 6 CONCLUSIONS

To date, this is the first study to assess sex differences comprehensively across physical strength and power, psychological traits of grit and resilience, as well as sleep quality following 61+ days of arduous military training under a multitude of stressors. Male and female Soldiers responded similarly to 61+ days of arduous military training. Both sexes experienced the same chronic stress exposure and high physical demands that led to marked declines in sleep and physical strength. Despite psychosocial stressors from simulated military operations and peer and instructor evaluations, non‐physical factors of grit and resilience remained stable from course start to finish for all participants completing the course. Frequent training in a multi‐stressor environment combined with chronic sleep loss may lead to cumulative deteriorations to physical strength and power without sex bias. Further understanding the response of individuals to extreme and prolonged training environments will aid in modernizing physical training and recovery to promote health prevention and resilience for every individual (Soldier).

## AUTHOR CONTRIBUTIONS

**Tyler E. Oliver:** Data curation; formal analysis; investigation; methodology; project administration; supervision; visualization. **Anna V. Oppenheimer:** Data curation; formal analysis; methodology; software; supervision; validation; visualization. **Emily S. Lange:** Data curation; formal analysis; visualization. **Samantha J. Goldenstein:** Data curation; formal analysis; investigation. **P. Matthew Bartlett:** Data curation; formal analysis; methodology; project administration. **Jess A. Gwin:** Conceptualization; data curation; formal analysis; investigation; methodology; supervision; validation. **Kristin J. Heaton:** Data curation; formal analysis; investigation; methodology; supervision. **Holly L. McClung:** Conceptualization; data curation; formal analysis; funding acquisition; investigation; methodology; project administration; resources; supervision.

## FUNDING INFORMATION

This study was funded by the Military Operational Medicine Research Program MO220015, United States. Army Medical Research and Materiel Command. Funders played no role in study design, execution, data interpretation, or dissemination of results.

## CONFLICTS OF INTEREST

The authors have no competing interests to declare.

## DISCLAIMER

The investigators have adhered to the policies for protection of human volunteers as prescribed in DODI 3216.02, and the research was conducted in adherence with the provisions of 32 CFR Part 219. 2. Citations of commercial organizations and trade names in this paper do not constitute an official Department of the Army endorsement or approval of the products or services of these organizations. This research was supported in part by an appointment of one of the researchers to the Department of War (DOW) Research Participation Program administered by the Oak Ridge Institute for Science and Education (ORISE) through an interagency agreement between the U.S. Department of Energy (DOE) and the DOW. The opinions or assertions contained herein are the private views of the authors and are not to be construed as official or as reflecting the views of the Army or the DOW, DOE, or ORAU/ORISE.

## Data Availability

Data described in the manuscript will not be made available because due release of data is considered PII and lack of consent by study participants for dissemination beyond immediate study use.
